# The Unsupervised Feature Selection Algorithms Based on Standard Deviation and Cosine Similarity for Genomic Data Analysis

**DOI:** 10.3389/fgene.2021.684100

**Published:** 2021-05-13

**Authors:** Juanying Xie, Mingzhao Wang, Shengquan Xu, Zhao Huang, Philip W. Grant

**Affiliations:** ^1^School of Computer Science, Shaanxi Normal University, Xi’an, China; ^2^College of Life Sciences, Shaanxi Normal University, Xi’an, China; ^3^Department of Computer Science, Swansea University, Swansea, United Kingdom

**Keywords:** unsupervised feature selection, gene selection, standard deviation, cosine similarity, 2-dimensional space

## Abstract

To tackle the challenges in genomic data analysis caused by their tens of thousands of dimensions while having a small number of examples and unbalanced examples between classes, the technique of unsupervised feature selection based on standard deviation and cosine similarity is proposed in this paper. We refer to this idea as SCFS (Standard deviation and Cosine similarity based Feature Selection). It defines the discernibility and independence of a feature to value its distinguishable capability between classes and its redundancy to other features, respectively. A 2-dimensional space is constructed using discernibility as x-axis and independence as y-axis to represent all features where the upper right corner features have both comparatively high discernibility and independence. The importance of a feature is defined as the product of its discernibility and its independence (i.e., the area of the rectangular enclosed by the feature’s coordinate lines and axes). The upper right corner features are by far the most important, comprising the optimal feature subset. Based on different definitions of independence using cosine similarity, there are three feature selection algorithms derived from SCFS. These are SCEFS (Standard deviation and Exponent Cosine similarity based Feature Selection), SCRFS (Standard deviation and Reciprocal Cosine similarity based Feature Selection) and SCAFS (Standard deviation and Anti-Cosine similarity based Feature Selection), respectively. The KNN and SVM classifiers are built based on the optimal feature subsets detected by these feature selection algorithms, respectively. The experimental results on 18 genomic datasets of cancers demonstrate that the proposed unsupervised feature selection algorithms SCEFS, SCRFS and SCAFS can detect the stable biomarkers with strong classification capability. This shows that the idea proposed in this paper is powerful. The functional analysis of these biomarkers show that the occurrence of the cancer is closely related to the biomarker gene regulation level. This fact will benefit cancer pathology research, drug development, early diagnosis, treatment and prevention.

## Introduction

The rapid development of high-throughput sequencing technology has produced a large amount of genomic data related to protein, gene and life metabolism. It has become a hot spot research field of life medicine to detect biomarkers and undertake related analyses using bioinformatics methods. It is known that the personal medicine program of United States of America and the precision medicine program in China were initiated in 2015 and 2016 respectively (Xie and Fan, 2017). More and more researchers have turned their attention to medical data analysis and to data-driven intelligent medical treatments using artificial intelligence techniques (Orringer et al., 2017; Esteva et al., 2017; Kim et al., 2018; Bychkov et al., 2018).

Cancers have become the main killer of humankind and there are seven persons diagnosed with cancers per minute in China in 2014 (Global Burden of Disease Cancer Collaboration, 2018; Cao and Chen, 2019). According to statistics by the IARC (International Agency for Research on Cancer) from WHO (World Health Organization) and GBD (Global Burden of Disease Cancer Collaboration), cancer cases increased by 28% between 2006 and 2016, and there will be 2.7 million new cancer cases emerging in 2030. Genomics data can reveal cancer related gene expression and regulation. There is a complex regulation network between genes. It has become popular to detect the biomarkers of cancers from the massive genomic data using the feature selection and classification techniques of machine learning (Xie and Gao, 2014; Xie et al., 2016b, 2020a,b; Esteva et al., 2017; Ye et al., 2017; Wang et al., 2017; Dong et al., 2018). The genomic data are usually of very high dimensions and small number of samples, and are always imbalanced, which lead to challenges for the available classification algorithms, especially with regard to the stability and generalization of the available algorithms (Diao and Vidyashankar, 2013). Feature selection algorithms can benefit the classification algorithms’ stability and generalization by selecting the key features related to cancers and eliminating the redundant and noisy features simultaneously (Ang et al., 2016; Dashtban and Balafar, 2017; Dong et al., 2018; Xie et al., 2019, 2020a,b).

Feature selection algorithm searches feature subsets from the search space composed of all combinations of features. It is an NP hard problem to detect the optimal feature subset (Fu et al., 1970). The common way is to use heuristics to find it. The feature subset is usually highly relevant to the classification problem and can improve the classification performance of the learning algorithm. Feature selection algorithms can be classified into Filters (Blum and Langley, 1997) or Wrappers (Kohavi and John, 1997) according to whether the feature selection process depends on the later learning algorithms or not. Filters are not dependent on the later learning algorithms while Wrappers are dependent, which lead to the fast efficiency of Filters and the time consuming load of Wrappers. However, wrappers can always detect the feature subset with high performance while with small number of features, but the limitations are that the feature subset can easily fall into overfitting with poor generalization. Therefore the hybrid feature selection algorithms have been studied and become the *ad hoc* research field in recent years (Xie and Wang, 2011; Kabir et al., 2011; Xie and Gao, 2014; Lu et al., 2017; Xie et al., 2019). Furthermore, feature selection algorithms can also be classified as supervised or unsupervised algorithms according to whether the class labels of training data are used or not in the feature selection process. Wrappers are always supervised feature selection algorithms while filters may be supervised, unsupervised or semi-supervised algorithms (Ang et al., 2016). Supervised feature selection algorithms usually realize feature selection by evaluating the correlation between features and class labels, such as mRMR (Minimal redundancy-maximal relevance) proposed by Peng et al. (2005). Supervised feature selection algorithms are always superior to semi-supervised and unsupervised feature selection algorithms in selecting powerful feature subsets due to its using the labels of samples. Semi-supervised feature selection algorithms are always deal with samples some of which having labels while others not, such as LRLS (Label reconstruction based laplacian score) proposed by Wang J. et al. (2013). The situation is that there are amount of data without class labels in the world and it is time-consuming or impossible to get labels for them. Therefore it is very important to study the unsupervised feature selection algorithms. However, the unsupervised feature selection problems are particularly difficult due to the absence of class labels that would guide search for relevant information. Even though, it has attracted many researchers to focus on this field, such as the feature entropy sorting based feature selection algorithm proposed by Dash et al. (1997). It adopted entropy to evaluate the importance of features to realize the unsupervised feature selection. Furthermore, Mitra et al. (2002) proposed the unsupervised feature selection algorithm based on their defined maximum information compression index to eliminate redundant features. Xu et al. (2012) proposed UFS-MI (Unsupervised feature selection approach based on mutual information). He et al. (2006) proposed the unsupervised feature selection algorithm based on manifold learning, and the importance of a feature is evaluated by its power of locality preserving, or, Laplacian Score. Zhao et al. (Zhao and Liu, 2007) proposed SPEC (Spectral analysis based feature selection) algorithm, which studied how to select features according to the structures of the graph induced from a set of pairwise instance similarity and employed the spectrum of the graph to measure feature relevance and elaborate how to realize spectral feature selection. As a result that the features which are consistent with the graph structure would comprise the optimal feature subset. Cai et al. (2010) proposed the MCFS (Multi-Cluster Feature Selection) algorithm, which selected those features to comprise the optimal feature subset such that the multi-cluster structure of the data can be best preserved by solving a sparse eigen-problem and a L1-regularized least squares problem. Hou et al. (2011) proposed a feature selection algorithm via joint embedding learning and sparse regression, which defined the weight using the locally linear approximation to construct graph and unified embedding learning and sparse regression to perform feature selection. Yang et al. (2011) proposed UDFS (Unsupervised discriminative feature selection) algorithm, which obtained the feature subset of the strong discriminant structure by maximizing the local inter-class divergence and minimizing local intra-class divergence simultaneously while minimizing the L2,1 norm of the coefficient matrix of the linear classifier. Li et al. (2012) proposed the NDFS (Non-negative discriminant feature selection) algorithm, which adopted spectral clustering to learn the cluster labels of the input samples while the feature selection is performed simultaneously. The joint learning of cluster labels and feature selection matrix enabled the NDFS algorithm to detect the most discriminative features. Qian et al. (Qian and Zhai, 2013) proposed an extended unsupervised feature selection algorithm named RUFS (Robust unsupervised feature selection). L2,1 norm minimization method was used in the process of label learning and feature selection to eliminate redundant and noisy features. Xie et al. (2018) proposed a distribution preserving feature selection (DPFS) method for unsupervised feature selection. Those features were selected which can preserve the distribution of the data. Liu et al. (2005) proposed a K-means based feature selection algorithm named as KFS, which performed supervised feature selection on several various clustering results of K-means to get the feature subset. Jiang et al. (2008) presented the CBFS (Clustering-based feature selection) algorithm, which defined the discriminative of each feature based on the difference between different clusters of each feature such that detecting the feature subset. Ling et al. (Ling and Ji, 2007) proposed a clustering ensemble based unsupervised feature selection algorithm by adopting a clustering algorithm to learn data labels and the ReliefF algorithm to perform feature selection. Wang et al. (Wang and Jiang, 2015) proposed unsupervised feature selection algorithm named FSFC (Feature selection method based on feature clustering), which defined the mean-similarity measure for each feature, then group all features into clusters, and select the representative feature from each cluster to comprise the feature subset. Panday et al. (2018) introduced two unsupervised feature selection algorithms by using a cluster-dependent feature-weighting mechanism to reflect the within-cluster degree of relevance of a specific feature. Features with a relatively high weight would comprise the feature subset. Xie et al. (2016a) put forward two unsupervised feature selection algorithms by defining the feature density and feature distance. The denser a feature, the more representative it is, and the more distant of a feature, the less is its redundancy. They adopted the product of the density and the distance of a feature to measure its contribution to the classification. He et al. (2017) proposed the unsupervised feature selection algorithm named DGFS (Decision graph-based feature selection). They defined the local density and the discriminant distance for a feature, and the decision score to evaluate the feature.

To summarize the aforementioned analyses we know that it is very challenging to analyze the genomic data, especially the gene expression data with tens to thousands dimensions while with very small number of samples. The worst thing is that this kind of data are always imbalanced and it is very difficult to get the class labels for the data. Therefore it is very difficult to find a stable and good generalization algorithm for analyzing this kind of genomic data.

To tackle this challenging task, this paper will focus on the feature selection problem for genomic data analysis under an unsupervised learning scenario. It will propose the unsupervised feature selection technique based on the standard deviation and the cosine similarity of variables. We refer to this as SCFS (unsupervised Feature Selection via Standard deviation and Cosine similarity scores of variables), which defines the feature discernibility and feature independence. The standard deviation of a feature is to define its discernibility while the cosine similarity is to define the independence or redundancy of a feature. Three unsupervised feature selection algorithms are derived from SCFS according to the various definitions of feature independence. These three unsupervised feature selection algorithms are SCEFS (Feature Selection via Standard deviation and Cosine similarity with Exponent), SCRFS (Feature Selection via Standard deviation and Cosine similarity with Reciprocal), and SCAFS (Feature Selection via Standard deviation and Anti-Cosine similarity), respectively.

To detect the features with both high discernibility and high independence from the original features easily, we display all features in the two dimensional space with discernibility as *x*-coordinate and independence as *y*-coordinate, such that these features centralize in the upper right corner while others in the bottom left corner. These upper right corner features comprise the optimal feature subset. The feature contribution to classification is quantified by the area of the rectangle enclosed by the feature coordinate lines and the coordinate axes, and called the feature score in this paper. Compared to other unsupervised feature selection algorithms, our proposed three unsupervised feature selection algorithms are simple in principles, and with low computational load, and the detected feature subset is sparse while representative.

We test these three unsupervised feature selection algorithms on 18 cancer genomic datasets. The proposed SCEFS, SCRFS and SCAFS can accurately detect the key biomarkers causing cancer diseases. These biomarkers are usually with rich classification information and strong stability. This study provides a base and clue for pathological research, drug development, early diagnosis, treatment and prevention of cancers.

## SCFS Algorithms

This section will introduce the proposed unsupervised feature selection algorithms in detail.

### Feature Discernibility

Given training dataset *D* ∈ *R*^*m*×*d*^, where *m* and *d* are the number of samples and the dimension of the data respectively. The features are represented as *f*_1_,*f*_2_,⋯,*f*_*i*_,⋯,*f*_*d*_, then *D* = [*f*_1_,*f*_2_,⋯,*f*_*i*_,⋯,*f*_*d*_], *f*_*i*_ ∈ *R^m^*,*i* = 1,⋯,*d*. The samples are *x*_1_,*x*_2_,⋯,*x*_*j*_,⋯,*x*_*m*_, and *D* = [*x*_1_;*x*_2_;⋯;*x*_*j*_;⋯;*x*_*m*_], *x*_*j*_ ∈ *R^d^*,*j* = 1,⋯,*m*.

#### Definition 1

Feature discernibility: The discernibility of feature *f*_*i*_, refers to its distinguishable capability between categories and is denoted by *dis*_*i*_. The standard deviation of a variable embodies its differences on all samples so the larger the standard deviation, the more differences the variable value has on all samples. Therefore the standard deviation of a feature can represent its distinguishable capability between categories. The discernibility *dis*_*i*_ of feature *f*_*i*_ is calculated in (1). The larger *dis*_*i*_, the more distinguishable capability the feature has, so contributes more to the classification.

(1) $$d\,i\,s_{i}=\sqrt{\frac{1}{m-1}\,\sum_{j=1}^{m}{\left(f_{j\,i}-\frac{1}{m}\,\sum_{j=1}^{m}f_{j\,i}\right)}^{2}}\,i=1,2,\cdots ,d;j=1,2,\cdots ,m$$

where, *f*_*ji*_ means the value of sample *j* on its feature *i*.

### Feature Independence

Feature selection aims to detect the features whose distinguishable capability is strong while the redundancy between them is less. We propose the feature independence definition to measure the redundancy between features. The independence of feature *f*_*i*_ is represented as *ind*_*i*_, which can be defined using the cosine similarities between features. To represent the redundancy between feature *f*_*i*_ and the other features, we define the cosine similarity matrix ***C*** in (2), which quantifies the similarity between feature *f*_*i*_ and other features. We define three types of feature independence in the following definitions (3) - (5).

(2) $$C={\left(c_{i\,j}\right)}_{d\times d},i,j=1,\cdots ,dc_{i\,j}=\frac{\left|f_{i}•f_{j}\right|}{∥f_{i}∥\times ∥f_{j}∥}.$$

#### Definition 2

Exponential feature independence: This type of feature independence is defined in (3).

(3) $$ind_{i}=\{\begin{array}{c} \exp\left(\overset{d}{\underset{k=1}{\max}}(-c_{ik})\right),\\ \exp\left(\underset{k:dis_{k}>dis_{i}}{\min}(-c_{ik})\right), \end{array}\;\begin{array}{c} i=\arg\;\max\;\{dis_{j}|j=1,...,d\};\\ \text{otherwise} \end{array}$$

#### Definition 3

Reciprocal feature independence: This type of feature independence is calculated in (4).

(4) $$i\,n\,d_{i}=\left\{ \begin{array}{cc} \max_{k=1}^{d}\left(\frac{1}{c_{i\,k}}\right), & i=\arg\max\left\{d\,i\,s_{j}|j=1,\cdots ,d\right\};\\ \min_{k:d\,i\,s_{k}>d\,i\,s_{i}}\left(\frac{1}{c_{i\,k}}\right), & \text{otherwise}. \end{array}\right.$$

#### Definition 4

Anti-similarity feature independence: This kind of feature independence is calculated in (5).

(5) $$i\,n\,d_{i}=\left\{ \begin{array}{c} \max_{k=1}^{d}\left(1-c_{i\,k}\right),i=\arg\max\left\{d\,i\,s_{j}|j=1,\cdots ,d\right\};\\ \min_{k:d\,i\,s_{k}>d\,i\,s_{i}}\left(1-c_{i\,k}\right),\text{otherwise}. \end{array}\right.$$

The definitions (3)-(5) guarantee that the feature *f*_*i*_ will have the maximal independence as far as possible once it has the maximal discernibility. Otherwise, its independence is quantified using the maximal cosine similarity between it and feature *f*_*k*_ whose discernibility is just higher, such that the independence embodies as low a redundancy as far as possible.

### Feature Score

The expected feature subset is the one whose features are strongly related to labels while the redundancy between features is very low (Peng et al., 2005; Ding and Peng, 2005). The discernibility definition (1) in section “Feature Discernibility” shows that the feature with strong distinguishable capability has a large discernibility. The independence definitions in section “Feature Independence” show that a feature with low redundancy has high independence. Therefore the optimal feature subset comprises the features with both high discernibility and high independence. To detect these features with both high discernibility and high independence, we display all features in the 2-dimensional space with discernibility as *x*-coordinate and independence as *y*-coordinate such that the upper right corner features are those with both relatively high discernibility and independence. These features comprise the optimal feature subset.

To quantify the contribution of a feature to classification, we introduce the feature score in (6) to measure the significance of the feature. The feature score is defined as the area of the rectangle enclosed by the feature coordinate lines and coordinate axes. From the aforementioned definitions, we know that the features with higher scores have strong discernibility and low redundancy. These features comprise the feature subset, which coincides with the original destination (Fu et al., 1970; Ding and Peng, 2005; Peng et al., 2005) of feature selection.

#### Definition 5

Feature score: Feature score of *f*_*i*_ is defined as

(6) $$s\,c\,o\,r\,e_{i}=d\,i\,s_{i}\times i\,n\,d_{i}$$

Definition (6) guarantees that feature *f*_*i*_ will have a high score when its discernibility and independence are both high implying the feature will benefit classification. Therefore selecting the features with high score as the feature subset satisfies the requirements of the optimal feature subset while guaranteeing the selected features’ discernibility is strong and the redundancy is low.

### Detailed Steps of SCFS

From the definitions of feature discernibility, feature independence, and feature score, we can display all features in 2-dimensional space, and select the upper right corner features to comprise the feature subset. Because these upper right corner features are far away from the other features, the feature selection process can be achieved automatically. In addition, three types of independences are used to develop three unsupervised feature selection algorithms named SCEFS, SCRFS, and SCAFS respectively. The pseudo code of our unsupervised feature selection algorithms SCEFS, SCRFS, SCAFS are presented below:

#### Input

Training data *D* ∈ *R*^*m*×*d*^, where *m* and *d* represent the number of samples and features respectively; number of selected features *k* and the original feature set ***F***.

#### Output

The selected feature subset ***S***.

**BEGIN**

S←Φ;

FOR *i* = 1 to *d* DO

    Calculate the feature discernibility *dis*_*i*_ of *f*_*i*_ using formula (1);

END of FOR

FOR each *f*_*i*_ ∈ *F* DO

    Calculate the feature independence *ind*_*i*_ of *f*_*i*_ using formula (3), (4) or (5);

    Calculate the feature score *score*_*i*_ using formula (6);

END of FOR

Sort features in descending order according to their scores;

Select top *k* features to comprise the feature subset ***S***.

**END**

### A Toy Case Study

In this subsection we will test the correctness of our proposed feature score, arbitrarily choosing SCEFS for illustration. We synthetically generate toy test data using two groups of mean and covariance matrices resulting in two categories of data with normal distributions. There are 20 samples in each category and each sample embodies 100 features.

**FIGURE 1:**
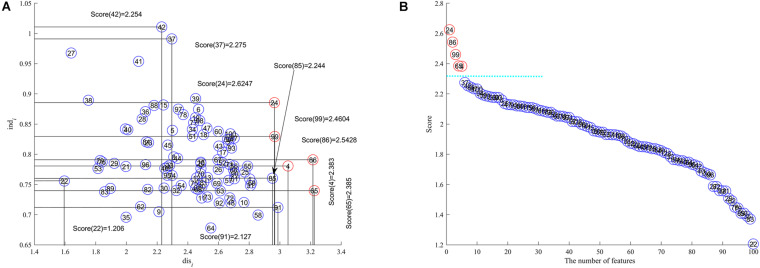
The toy case to test SCFS, **(A)** discernibility and independence are *x*-coordinate and *y*-coordinate respectively, **(B)** the number of features is the *x* axis and feature score the *y* axis respectively.

We adopt a bootstrap approach (Effron and Tibshirani, 1993; Kohavi, 1995) to partition the toy data into training and test subsets so that there are 28 (13 + 15) training samples and 12 (7 + 5) test samples. The feature discernibility, independence and score are calculated by using (1) and (3) and (6) respectively for the training data. All features are represented in 2-dimensional space with discernibility as the x axis and independence as y axis as shown in Figure 1A. In Figure 1B we display all features in descending order by their scores where the *x* axis is the number of features and the feature score is the y axis. The circled numbers in Figure 1 represents the feature ID in the toy data.

The results in Figure 1 show us that the features with IDs 24, 86, 99, 65, and 4 are the upper right corner features as their feature scores are higher than all others and is the feature subset we are trying to detect. Although features 37 and 42 have comparatively high independence, they do not have comparatively high discernibility; similarly with features 91 and 85, they have sufficiently high discernibility but comparatively low independence, so these four features are not selected for inclusion into the feature subset. The detected features are far away from other features because of their comparatively high scores, which is very clear from Figure 1B.

We test the classification capability of the detected features by building SVM classifiers using the SVM tool box LibSVM developed by Professor Lin et al. (Chang and Lin, 2011). The kernel function is a linear function, and the parameters are default except for the penalty factor *C* = 20. The results of the SVM classifiers achieved 100% accuracy when all the detected features 24, 86, 99, 65, and 4 are in the feature subset, while only 73.15% accuracy with only the top feature 24 in the feature subset, and 95.91% accuracy with the top 3 features 24,86, and 99 included.

Therefore the proposed SCFC method is valid in detecting the sparse and powerful feature subset whose features have comparatively high distinguishable capability and independence between each other so that a powerful classifier can be built using the feature subset.

### Complexity Analysis

Assume that there are *m* samples with *d* dimensions where it is usual that *d* > *m*, even *d*≫*m* always holds. The three proposed unsupervised feature selection algorithms SCEFS, SCRFS and SCAFS are all required to calculate the discernibility and independence for each feature. The time complexity of calculating discernibility is *O*(*dm*), and for independence is *O*(*d*^2^), and the time complexity to sort the feature scores is no more than *O*(*d*^2^). So, from the pseudo code in section “Detailed Steps of SCFS,” the total time complexity of all selection algorithms is *O*(*d*^2^). This is also the time complexity upper bound. The real consuming time may lower than this theoretical analysis by using matrix calculations embedded in MATLAB.

## Experiments and Analyses

As is well known genomic data analysis is very challenging in bioinformatics, especially gene expression data because this always has tens to thousands of dimensions while having very few samples and the data are always imbalanced. It is very difficult to find stable algorithms with good generalization for analyzing this kind of data.

This subsection will test the power of the unsupervised feature selection algorithms SCEFS, SCRFS, and SCAFS using high dimensional gene expression datasets of cancers. The detailed information of these data sets are shown in Table 1. The data sets of Gastric1 (accession: GSE29272), Gastric (accession: GSE37023), Non-small lung cancer (accession: GDS3627) and Prostate2 (accession: GDS2545) are from NCBI Gene Expression Omnibus (GEO) database^1^. The others are from Broad Institute Genome Data Analysis Center^2^ and Gene Expression Model Selector^3^.

**TABLE 1:** Descriptions of datasets.

Dataset name	Ng	Ns	Nc	Source
Colon	2000	62	2	Alon et al. (1999)
Leukemia	7129	72	2	Golub et al. (1999)
CNS	7129	90	2	Pomeroy et al. (2002)
CNS2	7129	60	2	Pomeroy et al. (2002)
DLBCL	7129	77	2	Shipp et al. (2002)
Lymphoma	4026	45	2	Alizadeh et al. (2000)
Carcinoma	7457	36	2	Notterman et al. (2001)
SRBCT	2308	83	4	Khan et al. (2001)
ALL1	12625	128	2	Chiaretti et al. (2004)
ALL4	12625	93	2	Chiaretti et al. (2004)
Lung cancer	12600	203	5	Bhattacharjee et al. (2001)
Prostate1	12625	102	2	Singh et al. (2002)
Prostate2	12558	108	3	Chandran et al. (2007)
11_Tumors	12533	174	11	Su et al. (2001)
Leukemia_MLL	12582	72	3	Armstrong et al. (2002)
Gastric	22645	65	2	Wu et al. (2012)
Gastric1	22283	144	2	Wang G. et al. (2013)
Non-small lung cancer	54675	58	2	Kuner et al. (2009)

*Note: Ng, Ns and Nc represent the number of features, instances and classes of dataset respectively.*

### Experiment Design and Evaluation Metrics

To test the power of our proposed SCEFS, SCRFS and SCAFS in detecting the optimal feature subsets for genomic data, we use them to find the feature subset of the 18 gene expression datasets shown in Table 1. Furthermore, we conduct comprehensive comparisons between their performances to that of other unsupervised feature selection algorithms, including EDPFS (unsupervised Feature Selection algorithm based on Exponential Density Peaks) (Xie et al., 2016a), RDPFS (unsupervised Feature Selection algorithm based on the Reciprocal Density Peaks) (Xie et al., 2016a), MCFS (Multi-Cluster Feature Selection) (Cai et al., 2010), Laplacian (Laplacian score for feature selection) (He et al., 2006), UDFS (Unsupervised Discriminative Feature Selection) (Yang et al., 2011), RUFS (Robust Unsupervised Feature Selection) (Qian and Zhai, 2013), NDFS (Non-negative Discriminant Feature Selection) (Li et al., 2012), and DGFS (Decision Graph-based Feature Selection) (He et al., 2017).

The compared algorithms EDPFS and RDPFS are our previously proposed unsupervised feature selection algorithms, which set the neighbors to be 2% when calculating the density of a feature. The algorithm DGFS set the cutoff distance *d*_*c*_ to the value of 2% of the total number of features, and sorted the feature distances in ascending order using Euclidean distance. The nearest neighbor number *K* of the compared algorithms MCFS, Laplacian, UDFS, RUFS and NDFS is set to 5. The similarity between features in Laplacian, RUFS and NDFS algorithms are cosine similarity, and the regularization parameter of UDFS and NDFS algorithms are set to 0.1

If there are missing values in the datasets, they are set to the intra-class mean. To avoid the impact from different scales of different features on experimental results due to the large differences among features of genomic data, the maximum and minimum standardization in (7) is used to normalize the data.

(7) $$f_{i,j}^{\prime }=\frac{f_{i,j}-\min\left(f_{•j}\right)}{\max\left(f_{•j}\right)-\min\left(f_{•j}\right)}$$

where *f*_*i,j*_ is the value of sample *i* on its feature *j*, *max*⁡(*f*_•*j*_) and *min*⁡(*f*_•*j*_) are the maximum and minimum value of feature *j* respectively.

Ten-fold cross validation experiments are carried out to test the power of the proposed unsupervised feature selection algorithms. Datasets are partitioned in the following way: the data are first shuffled randomly, and then each type of samples are put into 10 empty sample sets one by one, until each sample is allocated to a subset. Samples are divided into 10 folds evenly while avoiding the case that a fold does not contain samples from some types with small number of samples, especially in the imbalanced datasets. The nine folds comprise the training subset, and the remaining one fold is the test subset. The feature selection algorithms run on the training subset to detect the optimal feature subset, and the test subset is used to evaluate the detected feature subset. This process runs in turn until each fold is used as a test subset. To obtain the statistical experimental results, the above experimental process is run for five times, that is, the 10-fold cross validation experiments are run five times. The performance of a feature selection algorithm is evaluated using the mean classification results of the classifiers built on its selected feature subsets.

The code is implemented in MATLAB R2017b, and the experimental environment is Win10 64bit operating system, 192GB memory, Intel(R) Xeon(R) CPU E5-2666 v3@2.90GHz 2.90GHz (2 processors). The classifier adopts the SVM toolkit LibSVM developed by Lin et al. (Chang and Lin, 2011) and KNN embedded in MATLAB toolbox. The SVM classifier uses a linear kernel function with the penalty factor *C* = 20 and the default values for other parameters. The KNN classifier uses the nearest neighbor number *K* = 5. The unsupervised feature selection algorithms are evaluated in terms of the mean classification accuracy (simplified as Acc), AUC (MAUC for multi-class), F2-measure (referred to as F2) (Xie et al., 2019), Sensitivity, and Specificity of 10-fold cross validation experiments of their 5 runs. Where, F2-measure is proposed and defined for analyzing imbalanced data. It avoids the limits of F-measure which focuses on the positive class while ignoring the negative class. It is calculated by:

(8) $$F\,2-m\,e\,a\,s\,u\,r\,e=2*\frac{precision*\left(∼precision\right)}{p\,r\,e\,c\,i\,s\,i\,o\,n+\;\left(∼p\,r\,e\,c\,i\,s\,i\,o\,n\right)}$$

**FIGURE 2:**
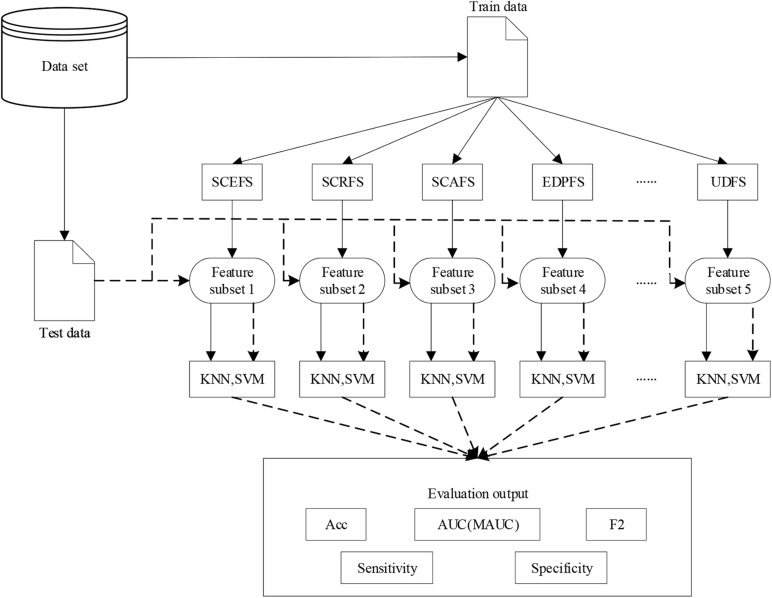
The flow chart of the experiments in this paper.

Where, precision and ∼precision are the ratios of the true positive and true negative samples recognized by the classifier to the positive and the negative samples recognized by the classifier, respectively. For multi-class *l*(*l* > 2) classification problem, we adopt one versus one method to transform the problem to be *l*(*l*−1)/2 binary classification problem. The F2 will be calculated using (9), similarly for Sensitivity and Specificity. Figure 2 shows the flow chart of the whole experiments in this paper.

(9) $$F_{2}=\frac{4}{l(l-1)}\sum_{i=1}^{l-1}\sum_{j=i+1}^{l}\frac{precision_{ij}*{\left(\sim precision\right)}_{ij}}{precision_{ij}+{\left(\sim precision\right)}_{ij}}$$

### Performance Comparison

This section will compare the performances of the proposed SCEFS, SCRFS, and SCAFS with other unsupervised feature selection algorithms EDPFS, RDPFS, MCFS, Laplacian, UDFS, RUFS, NDFS, and DGFS in selecting feature (gene) subsets on the gene expression datasets of cancers shown in Table 1. We first test the correctness of our defined feature score by comparing the proposed SCEFS, SCRFS, and SCAFS to the EDPFS, RDPFS, and DGFS algorithms on classic binary classification data Colon and multiclass classification data Leukemia_MLL. We evaluate the performances of the unsupervised feature selection algorithms in terms of Acc, AUC, F2, Sensitivity and Specificity of the classifier built using the feature subset detected by the algorithms according to feature scores.

#### Test of Feature Score

This subsection will test the proposed feature score by comparing the proposed SCEFS, SCRFS, and SCAFS with unsupervised feature selection algorithms EDPFS, RDPFS and DGFS. We display the features in 2-dimensional space by using the feature density (in EDPFS, RDPFS and DGFS), feature distance (in EDPFS, RDPFS and DGFS) and feature importance metric γ-score (in EDPFS and RDPFS), or decision graph score γ (in DGFS). It is similar to the proposed SCEFS, SCRFS, and SCAFS to display features in 2-dimensional space using feature independence as *y*-axis and feature discernibility as *x*-axis respectively, or display features in feature score descending order in 2-dimensional space using feature score as *y*-axis and the number of features as *x*-axis respectively.

**FIGURE 3:**
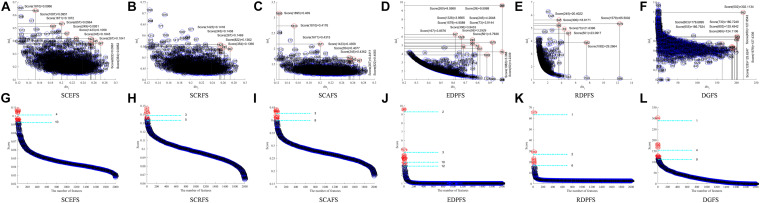
Features displaying in the 2-dimensional space of each algorithm on Colon dataset, **(A)** and **(G)** SCEFS, **(B)** and **(H)** SCRFS, **(C)** and **(I)** SCAFS, **(D)** and **(J)** EDPFS, **(E)** and **(K)** RDPFS, **(F)** and **(L)** DGFS.

**FIGURE 4:**
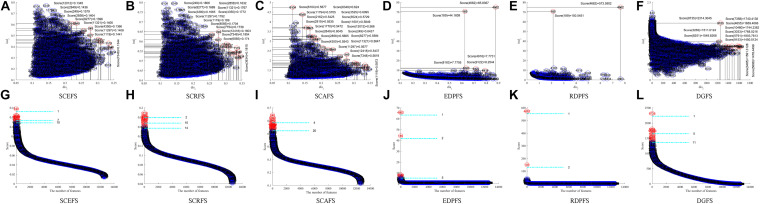
Features displaying in the 2-dimensional space of each algorithm on Leukemia_MLL dataset, **(A)** and **(G)** SCEFS, **(B)** and **(H)** SCRFS, **(C)** and **(I)** SCAFS, **(D)** and **(J)** EDPFS, **(E)** and **(K)** RDPFS, **(F)** and **(L)** DGFS.

Figure 3 shows the Colon cancer data features displayed in 2-dimensioanl space of the aforementioned six unsupervised feature selection algorithms. Table 2 shows the performances of the six feature selection algorithms in terms of Acc, AUC, F2, Sensitivity, and Specificity of the classifiers built using the detected feature subsets for Colon data. Figure 4 and Table 3 are the results of the aforementioned six feature selection algorithms on Leukemia_MLL dataset. The boldface font in Tables 2, 3 indicates the best results among the six algorithms.

**TABLE 2:** Performance comparison of KNN and SVM classifiers on Colon dataset by our algorithms and unsupervised feature selection algorithms based on density peaks.

Algorithms	KNN					SVM					Feature numbers
		
	Acc	AUC	F2	Sen	Spe	Acc	AUC	F2	Sen	Spe	
SCEFS	**0.840**	0.878	**0.817**	0.930	0.673	0.786	0.823	0.691	0.945	0.493	4
	0.824	**0.924**	0.768	**0.940**	0.610	0.795	0.814	0.716	0.940	0.527	10
SCRFS	0.821	0.873	0.809	0.910	0.660	0.757	0.784	0.539	0.970	0.363	3
	0.814	0.897	0.753	0.880	0.617	0.803	0.832	0.734	0.955	0.527	5
SCAFS	0.794	0.855	0.760	0.890	0.617	0.761	0.800	0.594	0.955	0.403	3
	0.834	0.894	0.809	0.920	**0.680**	0.805	0.827	0.745	0.940	0.560	8
EDPFS	0.614	0.779	0.246	0.850	0.180	0.648	0.716	0	**1**	0	2
	0.674	0.799	0.487	0.835	0.380	0.647	0.772	0.016	0.995	0.007	3
	0.811	0.886	0.788	0.890	0.670	**0.819**	0.853	**0.786**	0.935	**0.613**	10
	0.789	0.887	0.736	0.895	0.597	0.812	**0.855**	0.764	0.930	0.603	12
RDPFS	0.647	0.780	0.288	0.900	0.180	0.648	0.776	0	**1**	0	1
	0.647	0.780	0.288	0.900	0.180	0.644	0.776	0	0.995	0	2
	0.740	0.850	0.661	0.825	0.580	0.691	0.842	0.272	0.975	0.163	6
DGFS	0.551	0.698	0.164	0.790	0.11	0.648	0.738	0	**1**	0	1
	0.628	0.803	0.361	0.805	0.297	0.648	0.690	0	**1**	0	4
	0.601	0.763	0.346	0.765	0.303	0.648	0.743	0	**1**	0	9

**TABLE 3:** Performance comparison of KNN and SVM classifiers on Leukemia_MLL dataset by our algorithms and unsupervised feature selection based on density peaks.

Algorithms	KNN					SVM					Features numbers
		
	Acc	MAUC	F2	Sen	Spe	Acc	MAUC	F2	Sen	Spe	
SCEFS	0.642	0.841	0.539	0.672	0.719	0.624	0.883	0.397	0.564	0.637	1
	0.800	0.945	0.881	0.882	0.946	0.891	0.966	0.900	0.882	0.961	7
	0.803	**0.975**	**0.960**	**0.934**	**0.987**	**0.923**	**0.978**	**0.938**	0.920	**0.973**	10
SCRFS	0.466	0.764	0.424	0.591	0.606	0.410	0.773	0.058	0.128	0.634	2
	0.745	0.919	0.774	0.883	0.789	0.723	0.926	0.639	0.790	0.763	10
	0.721	0.919	0.757	0.901	0.754	0.752	0.949	0.764	0.914	0.769	14
SCAFS	0.719	0.896	0.684	0.798	0.779	0.719	0.918	0.595	0.739	0.780	4
	**0.824**	0.948	0.895	0.911	0.907	0.875	0.976	0.917	**0.940**	0.927	20
EDPFS	0.416	0.774	0.311	0.561	0.479	0.388	0.730	0	0	0.667	1
	0.477	0.765	0.422	0.644	0.549	0.388	0.713	0	0	0.667	2
	0.565	0.803	0.504	0.670	0.663	0.538	0.795	0.293	0.516	0.631	5
RDPFS	0.416	0.774	0.311	0.561	0.479	0.388	0.730	0	0	0.667	1
	0.477	0.765	0.422	0.644	0.549	0.388	0.713	0	0	0.667	2
DGFS	0.424	0.761	0.350	0.566	0.508	0.412	0.758	0.055	0.106	0.656	1
	0.606	0.846	0.528	0.670	0.702	0.606	0.828	0.285	0.596	0.632	5
	0.670	0.860	0.658	0.794	0.738	0.665	0.868	0.429	0.690	0.672	11

The experimental results in Figure 3 show that the proposed unsupervised feature selection algorithms SCEFS, SCRFS and SCAFS can detect two feature subsets of different scales for Colon dataset, while EDPFS, RDPFS and DGFS can detect three or four feature subsets. The number of features in each feature subset detected by our SCEFS, SCRFS and SCAFS ranges from 3 to 10, while EDPFS, RDPFS and DGFS detect from 1 to 12.

As can be seen from the experimental results in Table 2, the three proposed unsupervised feature selection algorithms are obviously better than the three compared algorithms EDPFS, RDPFS and DGFS when using KNN classifier. The performance of SCEFS algorithm is the best, and the performance of DGSF algorithm is the worst. However, our previously proposed EDPFS algorithm is better than the proposed SCEFS, SCRFS and SCAFS when using SVM classifier especially when the feature subset size is 10 or 12. The performance of SCEFS, SCRFS and SCAFS is similar, but it is obviously better than RDPFS and DGFS. Although EDPFS, RDPFS and DGFS obtain 100% sensitivity, especially DGFS whose sensitivities are all 100% no matter the feature subset comprise 1, 4 or 9 features, their corresponding F2 and specificity are both 0, which means that all normal people in the test subset are recognized as colon cancer patients using the detected feature subsets.

The results in Figure 4 show that the six unsupervised feature selection algorithms can detect the 2 or 3 feature subsets of different sizes for Leukemia_MLL dataset. The number of features is from 1 to 20. However, the EDPFS, RDPFS and DGFS algorithms can detect 2 or 3 feature subsets for Leukemia_MLL dataset. The number of features in these feature subsets is from 1 to 11.

As can be seen from results in Table 3, the proposed SCEFS can detect the optimal feature subset containing 10 features while having the best performance among the compared 6 unsupervised feature selection algorithms no matter whether using KNN or SVM classifier. It is obvious from the results in Table 3 that the proposed SCEFS, SCRFS and SCAFS outperformed the unsupervised feature selection algorithms EDPFS, RDPFS and DGFS.

To summarize the above analyses, we can assert that the proposed three unsupervised feature selection algorithms can detect the feature subset with strong discernibility having low redundancy. The detected feature subset usually comprises of a small number of features, and the classifiers built using the feature subset can obtain a good classification performance especially when the KNN classifier is used. Therefore the proposed SCEFS, SCRFS and SCAFS can realize a dimension reduction for high dimensional data meaning that our proposed feature score is powerful.

#### Comparison With Other Unsupervised Feature Selection Algorithms

**FIGURE 5:**
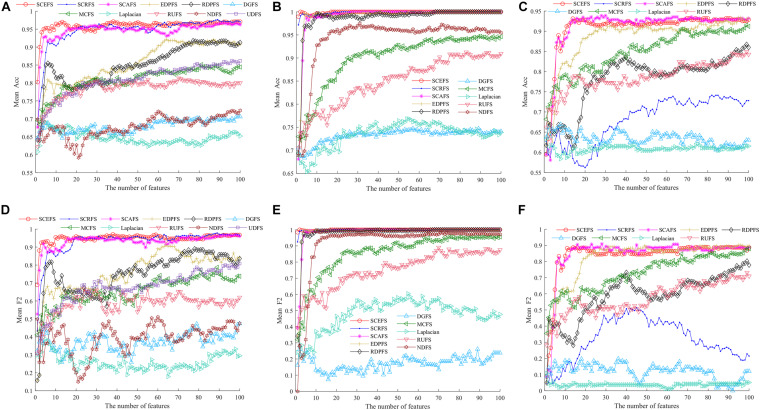
The average accuracy (Acc) and F2 of all algorithms on three datasets using KNN classifier, **(A)** and **(D)** Leukemia, **(B)** and **(E)** ALL1, **(C)** and **(F)** Non-small lung cancer.

**FIGURE 6:**
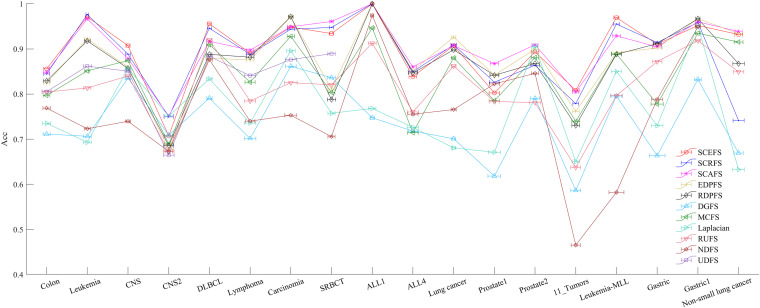
The maximal mean Acc comparison of each algorithm on 18 datasets using KNN classifier.

**FIGURE 7:**
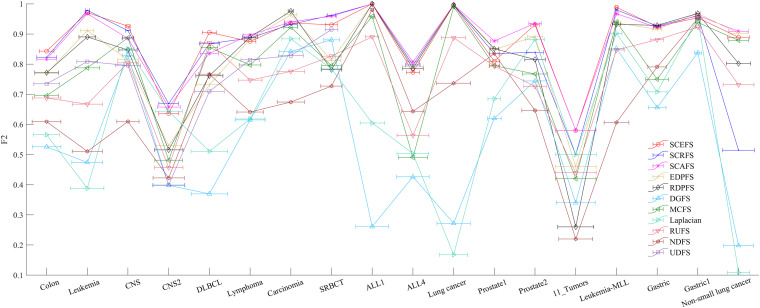
The maximal mean F2 comparison of each algorithm on 18 datasets using KNN classifier.

This subsection will compare the performance of our proposed SCEFS, SCRFS and SCAFS to that of the other set of eight unsupervised feature selection algorithms EDPFS, RDPFS, MCFS, Laplacian, UDFS, RUFS, NDFS and DGFS. We first show, in Figure 5, the performance of the above algorithms on three different scales of dimensions of datasets including Leukemia, ALL1 and Non-small lung cancer. Then we compare the performance of the above algorithms on the 18 datasets from Table 1 in Figure 6 and Table 4, and in Figure 7 and Table 5. The classifier used is KNN due to its simple and good performance in section “Test of Feature Score.” These 11 unsupervised feature selection algorithms are evaluated in terms of Acc and F2 of the KNN classifiers built using their detected feature subsets. We assume that the size of the feature subset is up to 100, that is, the feature subset consists of 100 detected features maximally. The NDFS and UDFS are so time consuming that we do not compare the algorithms to UDFS on the datasets with more than 10,000 features, nor for Non-small lung cancer dataset do we compare NDFS to other algorithms.

**TABLE 4:** The comparison between proposed algorithms and other algorithms in terms of win/draw/loss based on the maximal mean Acc.

Algorithms	SCEFS	SCRFS	SCAFS	EDPFS	RDPFS	DGFS	MCFS	Laplacian	RUFS	NDFS	UDFS
SCEFS	0/18/0	**9/1/8**	7/1/10	**10/1/7**	**12/1/5**	**17/0/1**	**18/0/0**	**16/0/2**	**18/0/0**	**17/0/1**	**18/0/0**
SCRFS	8/1/9	0/18/0	6/1/11	**10/1/7**	**12/1/5**	**18/0/0**	**16/0/2**	**16/0/2**	**17/0/1**	**18/0/0**	**18/0/0**
SCAFS	**10/1/7**	**11/1/6**	0/18/0	**14/1/3**	**14/1/3**	**18/0/0**	**18/0/0**	**17/0/1**	**17/0/1**	**18/0/0**	**18/0/0**

**TABLE 5:** The comparison between proposed algorithms and other compared algorithms in terms of win/draw/loss based on the maximal mean F2.

Algorithms	SCEFS	SCRFS	SCAFS	EDPFS	RDPFS	DGFS	MCFS	Laplacian	RUFS	NDFS	UDFS
SCEFS	0/18/0	**9/1/8**	6/2/10	**10/1/7**	**10/1/7**	**18/0/0**	**17/0/1**	**18/0/0**	**18/0/0**	**17/0/1**	**18/0/0**
SCRFS	8/1/9	0/18/0	8/1/9	**11/1/6**	**10/1/7**	**18/0/0**	**17/0/1**	**16/1/1**	**16/0/2**	**17/0/1**	**18/0/0**
SCAFS	**10/2/6**	**9/1/8**	0/18/0	**14/1/3**	**14/1/3**	**18/0/0**	**16/0/2**	**18/0/0**	**17/0/1**	**18/0/0**	**18/0/0**

Figure 5 shows the mean Acc and F2 on Leukemia, ALL 1 and Non-small Lung cancer datasets. Figure 6 shows the maximal mean Acc of each algorithm of its selecting feature subsets on 18 datasets from Table 1. Figure 7 displays the maximal mean F2 of each algorithm of its selecting feature subsets for 18 datasets from Table 1. The horizontal error bar at each data point in Figures 6, 7 indicates the standard deviation of the results of 5 runs of 10-fold cross validation experiments and the total error bar length is twice the standard deviation. Tables 4, 5 use the triplet of Win/Draw/Loss to evaluate the performance of the three proposed algorithms SCEFS, SCRFS and SCAFS with other unsupervised feature selection algorithms in terms Acc and F2 respectively. For example, for algorithms A and B, the 12/2/4 indicates that algorithm A is superior to algorithm B on 12 datasets, and equal to on 2 datasets, and inferior to on 4 datasets. We make 12/2/4 boldface to indicate that algorithm A defeats algorithm B in performance.

The results in Figure 5 show that the proposed SCEFS, SCRFS and SCAFS can detect feature subsets with good performance except for SCRFS on Non-small lung cancer dataset. The DGFS and Laplacian are the last two algorithms of the 11 compared unsupervised feature selection algorithms.

The results in Figures 5A,D show that the proposed SCEFS, SCRFS and SCAFS are superior to the other eight feature selection algorithms, especially SCEFS that performs best among the 11 feature selection algorithms. It can detect the feature subset containing 13 features which obtaining the Acc of 0.97and F2 of 0.96.

The results in Figures 5B,E on ALL1 dataset show that SCEFS and SCRFS algorithms perform very well when the feature subset comprises the top feature, and SCEFS can obtain the maximum Acc and F2 of 1 when selecting the top 2 features. Although SCAFS is not as good as SCEFS and SCRFS, it defeats the other compared feature selection algorithms and converges quickly with increasing features in the feature subset. Its KNN classifier can obtain Acc and F2 higher than 0.95 when there are top 4 features in the feature subset, and get the highest Acc and F2 of 1 when selecting the top 27 features in the feature subset. Our previously proposed EDPFS and RDPFS also perform well on ALL1 dataset, and can detect the feature subset classifying all samples correctly for the test subset.

The results in Figures 5C,F on Non-small lung cancer dataset show us that our proposed SCEFS and SCAFS are the top 2 feature selection algorithms among the 11 compared feature selection algorithms, especially SCAFS, which is the best. SCEFS and SCAFS outperform our previously proposed EDPFS. These three are superior to other compared feature selection algorithms. Our proposed SCRFS performs badly on Non-small lung cancer dataset. Its performance is just better than that of the feature selection algorithms DGFS and Laplacian.

The results in Figure 6 show us that the three proposed unsupervised feature selection algorithms SCEFS, SCRFS and SCAFS can detect the optimal feature subsets with best classification capability on nearly all datasets except for on the Carcinoma, Lung cancer and Gastric1 datasets. Our previously proposed EDPFS or RDPFS performs best on Carcinoma, Lung cancer and Gastric datasets. The performance of DGFS and Laplacian algorithms is poor. The results in Figure 6 also show us that the error bar of our three proposed algorithms is short on 18 datasets, which indicates that the proposed algorithms are more stable than the other 8 feature selection algorithms in 5 runs of 10-fold cross validation experiments. Therefore the proposed feature selection algorithms can detect the feature subset that has much more stable classification performance than that of other compared feature selection algorithms.

It can be seen from the results in Table 4 that the proposed SCAFS algorithm is the best, which can select the feature subsets with better classification performance than the algorithms DGFS, MCFS, NDFS and UDSF on 18 genomic data, and is superior to algorithms SCEFS and SCRFS on 10 and 11 data respectively. SCEFS is slightly better than SCRFS, and the former is better than the latter on 9 datasets. Although SCRFS is the worst among the proposed SCEFS, SCRFS, and SCAFS, it is superior to all the other 8 compared unsupervised feature selection algorithms EDPFS, RDPFS, DGFS, MCFS, Laplacian, RUFS, NDFS and UDFS.

The results in Figure 7 show that the proposed SCEFS, SCRFS and SCAFS perform best on most datasets except for on Carcinoma and Gastric1 datasets in terms of F2 of KNN classifiers built using the selected feature subsets. Our previously proposed RDPFS and EDPFS obtain the best performance on Carcinoma and Gastric1, followed by our proposed SCAFS, SCEFS and SCRFS algorithms. DGFS and Laplacian are the last two unsupervised feature selection algorithms among the overall 11 unsupervised feature selection algorithms. In addition, from the error bar of each algorithm for each dataset, it is clear that the proposed SCEFS, SCRFS and SCAFS can select the feature subset with strong stability. Therefore the proposed SCEFS, SCRFS and SCAFS are strong in finding powerful feature subsets.

The results in Table 5 show us that the proposed SCAFS is the best. It is superior to SCEFS and SCRFS on 10 and 9 datasets respectively, and equal to SCEFS and SCRFS on 2 and 1 datasets respectively. The proposed SCEFS ranks in the second place. Although SCRFS is inferior to SCAFS and SCEFS, it is superior to all the other eight compared unsupervised feature selection algorithms.

Summarizing the above analyses, it can be concluded that the proposed three unsupervised feature selection algorithms SCEFS, SCRFS and SCAFS are superior to our previously proposed EDPFS and RDPFS, and far superior to other compared feature selection algorithms. They can detect the feature subsets with good classification capability and strong stability. The KNN classifier built using the selected feature subsets obtain the expected performance on 18 cancer genomic datasets.

#### Statistical Significance Test of Algorithms

This subsection will undertake statistical tests on our proposed SCEFS, SCRFS and SCAFS, and the other compared unsupervised feature selection algorithms including EDPFS, RDPFS, DGFS, MCFS, Laplacian, RUFS, NDFS, and UDFS, to judge whether or not the results of our SCEFS, SCRFS and SCAFS are statistically significant. We adopt the Friedman’s test to discover the significant difference between the 11 unsupervised feature selection algorithms. If the significant difference has been detected by Friedman’s test, then the Nemenyi’s test is used as a *post hoc* test to see if there is significant difference between each pair of unsupervised feature selection algorithms. We conduct Friedman’s test at α = 0.05 using the results of each algorithm in terms of maximal mean Acc and F2 of KNN classifiers built using the selected feature subsets on 18 genomic datasets. If the null hypothesis that “all algorithms have the same performance” does not hold, then we adopt Nemenyi’s test to detect the significant difference between each pair of algorithms. We calculate the critical threshold *CD* in (10). If the difference of the mean ranks of a pair algorithm is greater than *CD*, then the null hypothesis that “the two algorithms have the same performance” is rejected, that is, the performances of the two algorithms are significantly different at the confidence degree of 1−α, that is 0.95; otherwise, the null hypothesis is accepted.

(10) $$C\,D=q_{\alpha }\,\sqrt{\frac{M\,\left(M+1\right)}{6\,N}}$$

In the above *M* and *N* are the number of algorithms and datasets respectively, and *q*_α_ can be found in textbook. For our Nemenyi’s test, *q*_α_ = *q*_0.05_ = 3.219, *M* = 11, *N* = 18, so *CD* = 3.5587.

At the statistical significance level of α = 0.05, the results of the Friedman’s test are here. For maximal mean Acc, *df* = 10, χ^2^ = 115.76, *p* = 3.652e-20; for maximal mean F2, *df* = 10, χ^2^ = 113.48, *p* = 1.058e-19. This Friedman’s test shows that *p* is much less than 0.05 no matter whether for Acc or F2, so we reject the null hypothesis that “all algorithms have the same performance” at the confidence degree of 0.95(= 1−α). We can say that there are strong significant differences between these 11 unsupervised feature selection algorithms.

**FIGURE 8:**
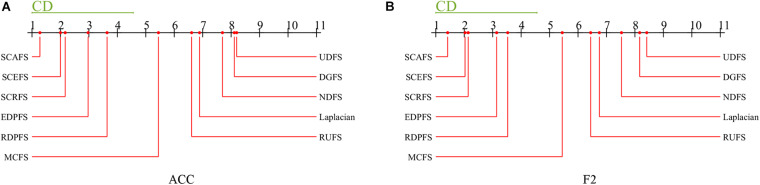
Comparison of unsupervised feature selection algorithms against each other on maximal mean Acc and F2 with Nemenyi’s test, **(A)** Acc, **(B)** F2.

Then as a *post hoc* test, the Nemenyi’s test is conducted to detect the significant difference between each pair of algorithms. The Nemenyi’s test results are shown in Figure 8.

The experimental results in Figure 8 show us that there is no significant difference between the three proposed unsupervised feature selection algorithms SCAFS, SCEFS, SCRFS in terms of the maximal mean Acc and F2, and there is also no significant difference between our SCAFS, SCEFS, SCRFS and our previously proposed algorithms EDPFS, RDPFS. However, there is significantly different between SCAFS, SCEFS, SCRFS, EDPFS, RDPFS, and MCFS, DGFS, UDFS, NDFS, Laplacian and RUFS algorithms. Our proposed SCAFS, SCEFS, SCRFS are better than the other eight unsupervised feature selection algorithms, especially better than MCFS, DGFS, UDFS, NDFS, Laplacian and RUFS algorithms. Our SCAFS is the best one among the 11 unsupervised feature selection algorithms.

### Run Time Comparison

This subsection chooses the five genomic datasets SRBCT, CNS, ProState2, Gastric and Non-Small Lung Cancer with very high dimensionalities to test the time performance of our three unsupervised feature selection algorithms SCAFS, SCEFS, SCRFS, while verifying the correctness of the theoretical time complexity analysis in section “Complexity Analysis.” All algorithms are run on the five datasets in 10-fold cross validation experiments for 5 runs. The average run time of each algorithm on five genomic datasets is compared with each other in Table 6.

**TABLE 6:** Runtime of each unsupervised feature selection algorithm on five datasets (in seconds).

Datasets	Algorithms										
	
	SCEFS	SCRFS	SCAFS	EDPFS	RDPFS	DGFS	MCFS	Laplacian	RUFS	NDFS	UDFS
SRBST	0.335 ± 0.53	0.244 ± 0.17	0.223 ± 0.10	0.590 ± 0.12	0.582 ± 0.13	0.413 ± 0.08	0.956 ± 0.75	0.027 ± 0.02	11.15 ± 5.50	13.56 ± 3.89	37.29 ± 8.92
CNS	1.617 ± 0.36	1.687 ± 0.53	1.611 ± 0.37	6.182 ± 0.95	6.220 ± 0.98	4.789 ± 0.79	2.975 ± 1.76	0.073 ± 0.02	17.85 ± 7.49	240.71 ± 22.15	1003.2 ± 43.63
Prostate2	5.478 ± 1.84	5.480 ± 1.43	5.900 ± 2.07	26.05 ± 9.28	24.19 ± 7.54	16.32 ± 4.76	2.871 ± 0.99	0.174 ± 0.05	43.10 ± 16.77	1851.4 ± 264.9	–
Gastric	12.49 ± 0.63	12.68 ± 0.95	12.63 ± 0.98	51.63 ± 4.26	51.71 ± 4.63	37.85 ± 2.82	2.412 ± 0.34	0.115 ± 0.02	40.71 ± 6.47	6957.0 ± 461.5	–
NLC	96.08 ± 9.59	95.50 ± 10.17	98.18 ± 12.83	756.97 ± 33.47	753.75 ± 40.19	353.86 ± 36.60	8.616 ± 1.59	0.350 ± 0.08	391.18 ± 28.30	–	–

*Note: NLC represents the Non-small lung cancer data.*

The results in Table 6 show that the Laplacian algorithm is the fastest one among the 11 unsupervised features selection algorithms on the five genomic datasets. It can complete feature selection in a short time. The proposed SCAFS, SCEFS, SCRFS feature selection algorithms have similar run times. They rank in second place after the Laplacian algorithm on SRBCT and CNS datasets with no more than 10,000 genes, and rank in the third place after Laplacian and MCFS algorithms on ProState2, Gastric and Non-Small Lung Cancer datasets which have more than 10,000 dimensions. They are definitely better than other compared unsupervised feature selection algorithms.

From the above analyses, we can say that although our proposed feature selection algorithms SCAFS, SCEFS, SCRFS are not the most efficient, their time consuming loads are acceptable on high dimensional datasets. They are faster than EDPFS, RDPFS, DGFS, RUFS, NDFS and UDFS algorithms when selecting optimal feature subsets on high dimensional datasets.

### The Bioinformatics Interpretation of the Selected Features of Our Algorithms

This subsection will take Prostate2 and Non-small lung cancer datasets as examples to conduct functional analysis on the genes selected by our SCEFS, SCRFS and SCAFS algorithms, and some of which may have known roles in cancer onset and development. Table 7 summarizes the gene biomarkers of Prostate2 and Non-small lung cancer detected by our SCEFS, SCRFS and SCAFS algorithms.

**TABLE 7:** The gene biomarkers of Prostate2 and Non-small lung cancer selected by our algorithms.

Datasets	Algorithms	Gene biomarkers
Prostate2	SCEFS	FOS, DNALI1, VWA5A, BTRC, PMF1-BGLAP, MGAT4C, KAT5, IER2, TRAF6, CYP27A1, CSPG4, MET, TIGR: HG3999-HT4269, LOC100289561, CDKN3, AP2B1, TK2, MSMB, TTPA, YME1L1, B3GALT2
	SCRFS	SEMG1, ALB, TNNT1, CRP, MYL1, CTNNB1, FGB, TNNC1, ACTA1, MYH7, MYLPF, CST4, FGG, HP, APOA1, DDN, MYL3, TPM2, FGA, SEMG2, NEB, SLN, APOC3, PCK1, ENO3, APOC4-APOC2
	SCAFS	CDKN3, FOS, CYP27A1, SSX2B, VWA5A, TTN, TGM4, CCL19, HPGD, CSPG4, AR, MSMB, TNNT1, MYL1, HDAC9, TNNI1, ALOX15B, PMF1-BGLAP, ACTA1, COL2A1, ACTC1, SERPINB5, PEG10, HBB
Non-small lung cancer	SCEFS	KRT5, SPRR1B, DSG3, DSC3, NTS, MAGEA6, MAGEA9B, XIST, SERPINB13, SPRR3, CLCA2, SPRR1A, MAGEA6, MAGEA10-MAGEA5
	SCRFS	GP2, RHOXF1, REG4, ACTN2, NCAN, PRL, REG1B, CYP2F1, FGF3, REG4, RHOXF2B, DEFA5, FRG2EP, GFI1B, BPIFB4, MUC6, EREG
	SCAFS	DSG3, NTS, XIST, SERPINB13, DSC3, SPRR1B, MAGEA9B, CLCA2, LIN28B, MAGEC2, SPRR3

The literature shows that many genes selected by our three unsupervised feature selection algorithms are associated with the prostate (He et al., 2013; Lu and Chen, 2015; Yu et al., 2015; Fajardo et al., 2016; Sjöblom et al., 2016) and non-small lung cancer (Wang et al., 2004; Monica et al., 2009; Agackiran et al., 2012; Sunaga et al., 2013; Argon et al., 2015; Tantai et al., 2015). For example, the gene MSMB selected by algorithms SCEFS and SCAFS is a key biomarker for prostate cancer (Kim et al., 2015; Sjöblom et al., 2016). The gene of MSMB is located in area 10q11.2 and the protein encoded is a member of the immunoglobulin binding factor family. The protein has inhibin-like activity and is one of the three most common proteins generated by the prostate. Several researches have shown the lower expression of MSMB protein in prostate cancer tissue and the cancer suppressive role in prostate cancer (Abrahamsson et al., 1988; Garde et al., 1999). The genes AR and MET are related to prostate cancer. They are selected by our SCAFS and SCEFS respectively. The gene AR is one of the most important genes in prostate cancer related genes. It has been amply demonstrated that AR gene regulation plays a key role in the survival mechanism of prostate cells (Balk and Knudsen, 2008; Fajardo et al., 2016). The increase of AR expression can reduce the content of prostate specific antigen in serum, and cause benign prostatic hyperplasia, and also has relation with the pathogenesis of prostate cancer. The gene MET participates in the biological processes of endothelial cell morphogenesis, signal transduction, cell surface receptor signaling pathway and cell proliferation. The MET signaling pathway plays an important role in cell migration, apoptosis, proliferation and differentiation, which can promote tumor cells to form more aggressive cell phenotype to avoid immunity and enhance the ability of tumor cells to survive, infiltrate and invade. The genes of KAT5, BTRC, FOS, CTNNB1, TGM4 and SERPINB5 detected by our algorithms have also been shown to be closely related to the occurrence and development of prostate cancer (Cao et al., 2013; He et al., 2013; Bernardo et al., 2015; Lu and Chen, 2015).

The genes DSC3, EREG, KRT5, LIN28B, NTS, XIST and DSG3 etc. selected by our three algorithms are closely connected with development of non-small lung cancer (Wang et al., 2004; Monica et al., 2009; Agackiran et al., 2012; Sunaga et al., 2013; Wen et al., 2014; Argon et al., 2015; Tantai et al., 2015). The gene DSC3 is the component of intercellular desmosome junctions, and involved in the biological processes of cell adhesion, protein stabilization and homophilic cell adhesion via plasma membrane adhesion molecules. Several studies demonstrated that DSC3 was a valuable biomarker for non-small lung cancer from other types of lung cancer (Agackiran et al., 2012; Masai et al., 2013). LIN28B is involved with regulation of transcription with DNA-templated, pre-miRNA processing, miRNA catabolic process and overexpressed in cancer cell lines and primary tumor of human. The gene LIN28B is known to be related to many types of diseases such as obesity, ovarian cancer and colon cancer (Leinonen et al., 2012; Pang et al., 2014; Lu et al., 2016). Recently published research has shown that LIN28B may affect the result of treatment of non-small lung cancer with radiotherapy, and may be biomarkers for non-small lung cancer (Wen et al., 2014).

Other gene biomarkers such as CDKN3 and SERPINB13 selected in this study may be worth the further prospective studies since they provide the best performance of classification for prostate cancer and non-small lung cancer datasets.

## Conclusion

This paper presented the unsupervised feature selection algorithms SCEFS, SCRFS, and SCAFS based on feature standard deviation and cosine similarity for tackling the challenges in cancer genomic data analysis. Feature discernibility is proposed and defined using its standard deviation, and also feature independence by cosine similarity. All features are scattered in 2-dimesional space using discernibility as *x*-axis and independence as y-axis respectively, so that the upper right corner features have both high discernibility and independence, and comprise the optimal feature subset. The feature score is proposed and defined as the area of the rectangle enclosed by the feature coordinate lines and coordinate axes, so as to quantify the contributions of the upper right corner features to classification. The theoretical analysis and the comprehensive experiments on 18 genomic datasets demonstrate that the proposed three unsupervised feature selection algorithms can detect the optimal feature subsets enclosing sparse and strong discernibility while having low redundancy features. The detected features by our proposed feature selection algorithms are most important biomarkers whose regulation levels are closely related to pathogeneses of cancers. This study provides a base for cancer pathological research, drug development, cancer early diagnosis, treatment and prevention.

## Data Availability Statement

The original contributions presented in the study are included in the article/supplementary material, further inquiries can be directed to the corresponding author/s.

## Author Contributions

JX made substantial contributions to the conception and revised the work. MW implemented all algorithms and wrote the experimental results. PG read through and revised the manuscript. JX, MW, SX, ZH, and PG discussed and designed this study. All authors read and approved the final manuscript.

## Conflict of Interest

The authors declare that the research was conducted in the absence of any commercial or financial relationships that could be construed as a potential conflict of interest.

## Footnotes

1 https://www.ncbi.nlm.nih.gov/geo/

2 http://portals.broadinstitute.org/cgi-bin/cancer/datasets.cgi

3 http://www.gems-system.org/
